# Elucidating the Intricate Roles of Gut and Breast Microbiomes in Breast Cancer Metastasis to the Bone

**DOI:** 10.1002/cnr2.70005

**Published:** 2024-08-26

**Authors:** Amruta Naik, Mukul S. Godbole

**Affiliations:** ^1^ Department of Biosciences and Technology, School of Science and Environmental Studies Dr. Vishwanath Karad MIT World Peace University Pune India

**Keywords:** bone, breast cancer, gut, metastasis, microbiome

## Abstract

**Background:**

Breast cancer is the most predominant and heterogeneous cancer in women. Moreover, breast cancer has a high prevalence to metastasize to distant organs, such as the brain, lungs, and bones. Patients with breast cancer metastasis to the bones have poor overall and relapse‐free survival. Moreover, treatment using chemotherapy and immunotherapy is ineffective in preventing or reducing cancer metastasis.

**Recent Findings:**

Microorganisms residing in the gut and breast, termed as the resident microbiome, have a significant influence on the formation and progression of breast cancer. Recent studies have identified some microorganisms that induce breast cancer metastasis to the bone. These organisms utilize multiple mechanisms, including induction of epithelial–mesenchymal transition, steroid hormone metabolism, immune modification, bone remodeling, and secretion of microbial products that alter tumor microenvironment, and enhance propensity of breast cancer cells to metastasize. However, their involvement makes these microorganisms suitable as novel therapeutic targets. Thus, studies are underway to prevent and reduce breast cancer metastasis to distant organs, including the bone, using chemotherapeutic or immunotherapeutic drugs, along with probiotics, antibiotics or fecal microbiota transplantation.

**Conclusions:**

The present review describes association of gut and breast microbiomes with bone metastases. We have elaborated on the mechanisms utilized by breast and gut microbiomes that induce breast cancer metastasis, especially to the bone. The review also highlights the current treatment options that may target both the microbiomes for preventing or reducing breast cancer metastases. Finally, we have specified the necessity of maintaining a diverse gut microbiome to prevent dysbiosis, which otherwise may induce breast carcinogenesis and metastasis especially to the bone. The review may facilitate more detailed investigations of the causal associations between these microbiomes and bone metastases. Moreover, the potential treatment options described in the review may promote discussions and research on the modes to improve survival of patients with breast cancer by targeting the gut and breast microbiomes.

## 1 Introduction

Metastasis, the dissemination of cancer cells from primary site to distant locations, is the leading cause of deaths in patients afflicted with cancer [1]. Cancers, as per the seed–soil hypothesis [2], tend to metastasize and establish in particular organs of the body [3]. For instance, breast cancer has approximately 70% propensity to metastasize to the bones [4]. Of which, luminal types preferentially metastasize to the bones in ≥70% of the cases [5]. Moreover, clinically, breast cancers tend to first metastasize to the bones [6], and patients with metastatic breast cancer tend to have poor survival outcomes [7]. Interestingly, patients with breast cancer with untreated osteoporosis or those with arthritis show a high tendency to develop bone metastasis [8, 9]; this indicates that priming of the bone architecture due to osteoporosis may serve as a favorable mechanism for establishment of metastasis in the bone. Moreover, studies have described the mechanisms by which breast cancer metastasizes to the bone and how bone serves as a supportive environment for breast cancer [10, 11]. However, whether factors other than breast cancer cells and bone microenvironment influence breast cancer metastasis to the bone warrant detailed investigation.

A seminal study by Bullman et al. showed the association of *Fusobacterium* with primary and metastatic colorectal cancer [12]. Specifically, *Fusobacterium* metastasized with colorectal cancer cells to the liver. The study suggested that *Fusobacterium* persisted even in xenografts derived from patient samples and occurred within vesicle‐like structures in cancer cells. The analyses highlighted the intrinsic and essential role of microbiota in the cancer microenvironment [12]. Further, in case of breast cancer, Banerjee et al. have shown a subtype‐specific enrichment of microorganisms in the breast tissue of patients with breast cancer [13, 14]. Next, Fu et al. showed that bacterial density is higher in the breast tumor tissue than in normal breast, with significantly different microbial communities and low alpha diversity [15]. They also showed the specific intracellular, and low extracellular, presence of microbes in the cancer cells. Moreover, Fu et al. showed that metastatic breast cancer cells harboring bacteria have a better survival ability than those without bacteria [15]. Taken together, studies have indicated that the breast tissue and gut microbiomes may influence the development, maintenance, or progression of breast and other cancers.

Treatment of metastatic cancers differs from that of primary cancer as they accumulate divergent mutations and mechanisms for their metastasis and establishment at the secondary site. For example, metastatic cancers tend to show high therapy resistance and recurrence [3, 5, 16]. Interestingly, Wenhui et al. found that gut microbial diversity decreases with breast cancer progression, and is lowest in patients with breast cancer metastasis to the bone [17]. Therefore, patient‐centric treatment strategies for targeting breast cancer metastasis are required, including exploiting the involvement of gut and breast microbiome in breast cancer. To facilitate discussion and research in this direction, here we have reviewed the current understanding of mechanisms by which microbes residing in the gut and breast tissue influence the development of primary breast cancer and metastasis of breast cancer to the bone. Additionally, we have summarized potential treatment strategies to impede breast cancer metastasis to the bone.

## 2 Overview of Gut and Breast Microbiome and Their Functions

The human body harbors a vast array of microorganisms, collectively known as microbiome, that play important role in maintenance of overall health [18]. The human microbiome outnumbers human cells in the body and the ratio of bacteria to human cells is approximately 10:1. Recent research has highlighted two important microbial communities—the gut and breast microbiomes [19]. These microorganisms encode over three million genes producing thousands of metabolites that significantly influence the physiological functions of the human body [20]. Disruption of gut microbiota due to factors, such as surgery or treatment with broad‐spectrum antibiotics, can affect the essential functions of human body and overall health [21]. Moreover, the breast microbiome may influence mammary gland development, lactation, and breast health, and may provide favorable environment for the development of benign and malignant tumors [19, 22].

The interactions between the human tissue and gastrointestinal (GI) tract or breast microbiota have been explored using metagenomic sequencing technologies, shotgun metagenomics and 16*S* rRNA sequencing for gene function analysis [23]. The symbiotic relationship between gut microbiome and human host is fundamental, as these microorganisms perform several essential functions, such as vitamin production, essential for human health. Additionally, they produce short‐chain fatty acids (SCFAs) through the fermentation of dietary fibers that provide energy to intestinal cells and help maintain gut health. Furthermore, gut microbiome aids in the breakdown of complex carbohydrates, fibers, and other dietary components that human enzymes cannot digest, and thus, facilitates nutrient absorption [24]. Gut microbiome also interacts with the immune system, where it plays a crucial role in maintaining immune homeostasis and defending against pathogens [25]. Thus, a multitude of functions of the microbiome aids in maintaining overall homeostasis in the body.

Furthermore, studies suggest that the gut microbiome is involved in regulating host metabolism [26]. For instance, imbalances in the gut microbiome have been linked to metabolic disorders such as obesity, chronic autoimmune disorders (multiple sclerosis, rheumatoid arthritis, type 1 diabetes, systemic lupus erythematous), nervous system disorders (Alzheimer's disease, Parkinson's disease), and respiratory syndrome (asthma, chronic obstructive pulmonary disease, and cystic fibrosis) [27, 28, 29]. The gut microbiome contributes to maintaining the integrity of the intestinal barrier, preventing invasion by harmful bacteria and pathogens. However, dysbiosis due to changes in eating habits, medications, or infections leads to the development of several disorders, including cancer [30].

The breast microbiome is less well‐studied than the gut microbiome, but emerging evidence suggests that it has important functions in providing protection against potentially harmful pathogens. For instance, the breast microbiome may play a role in shaping the infant's gut microbiome and immune system development [31]. Similar to the gut microbiome, the breast microbiome interacts with the immune system, potentially modulating immune responses and promoting immune tolerance [32]. Nevertheless, our understanding of the functions of the microbiome continues to evolve. Overall, the gut and breast microbiomes have multifaceted functions that extend beyond maintenance of normal tissue homeostasis and protection from pathogenic infections.

## 3 Interplay Between the Gut and Breast Microbiomes and Breast Cancer

First, we will discuss the association of gut microbiome with breast cancer. The gut microbiome composition influences immune function, metabolism, and inflammation, which are all relevant to carcinogenesis and cancer progression. Certain gut bacteria may influence the metabolism of estrogen, a hormone associated with breast cancer, for example, altered estrogen metabolism may affect breast tissue architecture and contribute to carcinogenesis [33, 34]. The role of “estrobolome,” a consortium of enteric microorganisms and their gene products capable of metabolizing estrogen and its derivatives, has been extensively studied. An enhanced activity of the estrobolome leads to elevated levels of circulating estrogen and its metabolites, increasing the risk of breast cancer [35]. Estrogen is metabolized by microorganisms producing β‐glucuronidase, which belong to the Clostridia and Ruminococcaceae families and members of Proteobacteria phylum [36, 37]. Interestingly, postmenopausal hormonal therapy, comprising estrogen‐like compounds, has been shown to increase the risk of breast and endometrial cancers. Therefore, clinical studies are underway to elucidate the effects of combinatorial hormone‐replenishment therapy (estrogen + progesterone biosimilars) on bone disorders and breast cancer risk in postmenopausal women [38]. We have previously reviewed the diverse modes by which gut microbiome can alter steroid hormone levels and activity in women. Moreover, we have described the ability of gut microbiome to modulate the anticancer effects of chemotherapeutic and immunotherapeutic agents [39]. An increase in circulating levels of estrogen and testosterone has been linked with breast cancer risk [40], and thus, enhanced biosynthesis of these hormones may influence growth and metastases of breast cancer as well.

Additionally, Li et al. have described that the composition of gut microbiome may modulate systemic inflammation and immune responses that influence the potential risk of developing breast cancer (NCT03358511) [41]. Further, animal experiments have highlighted the impact of gut microbiota alterations on tumor growth and treatment responses—different mice strains show varied responses to anticancer treatments. For example, Sivan et al. used a mouse model of melanoma and noted that different mice strains show varied rates of tumor growth and that tumors respond more effectively to some anticancer treatments, such as anti‐PD‐L1 [42]. Routy et al. transplanted patients' fecal microbiota into antibiotic‐treated mice or germ‐free mice and noted responses of mice to PD‐1 blockade [43]. Fecal microbiota transplantation (FMT) from patients with epithelial carcinomas who responded to PD‐1 blockade into germ‐free/antibiotic‐treated mice ensured that the mice too responded to PD‐1 blockade and showed tumor reduction. Interestingly, mice that received FMT from nonresponding patients showed restoration of response to PD‐1 blockade after oral supplementation with *Akkermansia muciniphila* [43]. Furthermore, detailed investigations by other groups on the composition of gut microbiota among patients with breast cancer with different clinical characteristics revealed that the absolute numbers of *Bifidobacterium* and *Blautia*, and proportions of *Faecalibacterium prausnitzii* and *Blautia* varied according to clinical stages of breast cancer [44, 45].

Second, the breast was traditionally considered as a sterile organ. However, recent studies have shown that microorganisms that reside in the breast tissue and ductal fluid can significantly contribute to the development of breast cancer [46, 47]. Furthermore, significant differences in the breast microbiome composition between women with and without breast cancer have been observed, and that these differences include varied abundance of specific bacterial species, such as *Escherichia coli* [32]. A study by Xuan et al. reported the varied abundance of *Methylobacterium radiotolerans* and *Sphingomonas yanoikuyae* between paired healthy and tumor tissues, suggesting involvement of these microbes in cancer development [48]. Similarly, Hieken et al. highlighted notable differences in the bacterial profiles in the breast tissue of healthy individuals and patients with breast cancer [22]. A study by Pawlik et al. observed significantly higher relative abundance of *Bacillus*, *Staphylococcus*, Enterobacteriaceae, Comamonadaceae, and *Bacteroidetes* in patients with breast cancer than those without breast cancer. In contrast, they observed that a reduction in the levels of lactic acid bacteria with known anticarcinogenic properties. Interestingly, nipple aspirate fluid collected from patients with breast cancer had a significantly different microbiota profile than that collected from healthy individuals. Pawlik et al. also found *Alistipes* to be relatively abundant in the nipple aspirate fluid collected from patients with breast cancer [49]. Chan et al. characterized the breast ductal microbiota from women with breast cancer and healthy controls. Firmicutes, Proteobacteria, and Bacteroidetes were found to be the most abundant bacterial phyla in the breasts of patients with ductal carcinoma [31]. Furthermore, in addition to the presence of signatures for bacteria, parasites, and fungi, Banerjee et al. observed a substantial presence of viruses in the breast tumor tissue or tumor microenvironment, this suggests the association of viral signatures with specific breast cancer subtypes. They also suggested microbial signatures unique to the four subtypes of breast cancer. Various bacterial phyla have been found to dominate the breast microbiota of patients with ductal carcinoma, and the presence of microbial signatures, including viruses, has been associated with specific breast cancer subtypes [13]. However, establishing a causal relationship between these alterations in the breast microbiome and breast cancer development requires further in‐depth research to uncover precise connections and underlying mechanisms.

## 4 Microbiomes Influence Bone Architecture and Immune Responses to Alter Tumor Metastasis

### 4.1 Effects of Gut Microbiome on Bone Architecture

Bones provide a unique microenvironment, such as the bone matrix, osteoblasts, and osteoclasts, which supports the growth and proliferation of cancer cells. Certain factors released by cancer cells also lead to either bone resorption or bone formation [50]. Previous investigations on human subjects and animal models have reported the regulatory involvement of gut microbiome to maintain the composition of bone, bone mineral density, postnatal skeletal development, and development of degenerative bone disorders such as osteoporosis [51, 52, 53]. These studies have also indicated that alterations in the gut microbiome composition influence systemic inflammation and immune response that may enhance the risk of cancer development and progression [54]. Particularly, the gut microbiome may influence the tumor microenvironment, immune responses, and bone metabolism that may affect the likelihood and progression of bone metastases [55]. Moreover, Sjögren et al. explained how absence of microbes in the gut leads to enhanced bone mass of the trabecular bone due to reduction in the number of osteoclasts, along with reduction in the levels of CD4^+^ T cell osteoclast precursors in the bone marrow. They showed that low levels of TNFα in the colon and bone marrow of germ‐free mice correlate with reduced osteoclastogenesis, and hence, the low numbers of osteoclasts in the trabecular bone [56]. Further, certain products of gut microbiome show myriad effects on cancer cells and bone homeostasis, such as enhancing osteoclast activity at the bone metastatic site, which aids in forming suitable microenvironment for cancer cells at bone surfaces. For example, Lucas et al. described that SCFAs, produced during intestinal fermentation, increase bone mass via enhanced intestinal absorption of calcium. Specific components—propionate and butyrate—were found to reduce expression of *TRAF6* and *NFATc1* and inhibit osteoclast differentiation and bone resorption [57]. Whereas, the effects of serotonin, folic acid, and polyamines—products of gut microbiome—on bone health have been reviewed elsewhere [58].

Next, Sjögren et al. have also described the role of gut microbiome in regulating intestinal hormones that control osteoblast formation and activity, regulating steroid hormone levels, calcium absorption, bone mineral density, and so on [56]. Certain prebiotic and probiotic strains have been shown to promote calcium uptake and bone mineralization [59]. Probiotic strains with anti‐inflammatory properties have been shown to confer protection against ovariectomy‐induced bone loss in rodents [60, 61, 62]. Further, a randomized, multicenter clinical trial conducted by Curiac et al. suggested that probiotic treatment with a combination of *Lactobacillus* strains reduced lumbar spine bone mineral density in early postmenopausal women [63]. A similar clinical trial by Nilsson et al. suggested that treatment with *Lactobacillus reuteri* leads to reduction in bone loss in elderly women with low bone density [64]. These results warrant long‐term clinical validations to systematically analyze the effects of probiotic treatment at specific bone sites in postmenopausal women. Interestingly, mice devoid of gut microbiome have been shown to be spared from bone loss due to deficiency of sex steroid hormones [65]. This highlights the role of gut microbiome in altering the integrity of bones, and suggests that an unfavorable microbial composition may exacerbate bone loss due to inflammatory immune responses, especially in postmenopausal women devoid of estrogen [66].

### 4.2 Effects of Microbiome on Functioning of Immune System

Several studies have explained that gut microbiome regulates the functioning of immune system. First, Khosravi et al. showed that germ‐free mice tend to have lower levels of neutrophils and monocytes in their bone marrow, possibly due to a decrease in the levels of granulocyte–monocyte progenitors. Recolonization of these mice with microbial cocktail was found to improve myelopoiesis and prevent systemic infection by *Listeria monocytogenes* [67]. Second, Charles, Ermann, and Aliprantis have reviewed how intestinal dysbiotic states alter the T‐cell mechanisms that negatively impact bone health [68]. Arthritic mice show increase in the levels of mast cells in the bone that serve as a chemoattractant for breast cancer cells. Breast cancer cells modulate the mast cell population in the bone upon successful establishment. However, nonarthritic mice have been found to show low infiltration of mast cells in the primary and metastatic breast cancers [69]. Additionally, a study by Rhee, Pothoulakis, and Mayer suggested that enhanced osteoclast activity may increase the space available for establishment of metastases. The gut microbiota interacts with the intestinal endocrine cells, and thus, influences their hormone‐secretion patterns. This affects the direct communication with the visceral afferent nerves and immune cells in the host [70]. Interestingly, germ‐free mice have immature mucosal immune systems and the Peyer's patch contain low levels of germinal centers and IgA‐producing plasma cells and lamina propria CD4^+^ T cells [71]. Studies suggested that the gut microbiota can also aid in shaping systemic immunity by regulating the number of CD4^+^ T cells and germinal centers in the spleen [72, 73]. Specifically, Mazmanian et al. showed that a polysaccharide of *Bacteroides fragilis* orchestrates maturation of the immune system, including optimization of T_H_1/T_H_2 balances and directing lymphoid organogenesis [73]. Taken together, these studies clarify that the gut microbiota can influence the outcomes of local and distant organs, and even the overall immune responses, which together influence distant metastases.

## 5 Mechanisms Governed by Gut and Breast Microbiomes in Regulating Breast Cancer Metastases to Bones

### 5.1 Gut and Breast Microbiomes InfluenceBreast Cancer Metastasis

Bones serve as a preferable site for metastasis of several cancer types, including breast cancer, lung cancer, prostate cancer, and head and neck cancer [3]. For instance, a study by Pal et al. explains how gut microbiome increases expansion of intestinal natural killer cells and Th1 cells in the bone, and thus, restrains growth of melanoma cells in the bone [74]. Alterations in the microbiome have been associated with increased inflammation and changes in immune responses that can contribute to cancer development and progression. Specifically, bacteria in the human gut have been found to influence the activity of distant organs in multiple ways, as reviewed by Hernandez et al. [75]. First, bacteria can translocate through the circulatory system, especially in a diseased state due to increase in intestinal permeability. While a majority of the translocating bacteria are destroyed by the immune system, certain microbial‐associated molecular patterns (MAMPs), such as lipopolysaccharide, peptidoglycan, flagellin, and cell‐free DNA, which are released from the dead bacteria, remain in circulation [76]. Second, bacteria in the gut can directly release MAMPs into circulation; these molecules can activate innate or adaptive immune responses. For instance, in the bone, MAMPs can influence bone remodeling by stimulating toll‐like receptors (TLRs), such as TLR2, TLR4, and TLR5 [77, 78, 79, 80]. In case of breast cancer, while the precise mechanisms and interactions are not yet fully delineated, there is growing evidence that suggests the contribution of gut and breast microbiomes in influencing breast cancer metastasis to distant organs and tissues, such as the bone [81, 82]. For instance, Parhi et al. showed that Fap2 protein of *Fusobacterium nucleatum* recognizes Gal‐GalNAc on breast tumor cells and helps *F. nucleatum* to colonize tumor tissue. They showed that bacterial colonization led to progression and metastasis of breast cancer cells, possibly via depletion of T cells in the tumor microenvironment [83]. In contrast, a study by Bernardo et al. suggested that antibiotic‐induced suppression of *Staphylococcus epidermis* in the breast tissue microbiota decreased breast cancer aggressiveness and induced antitumor immune response [84]. While it has been suggested that anticancer treatment comprising antibiotics may improve therapy outcomes, studies have described the potentially detrimental effects of antibiotics [21, 85]. Thus, to achieve balance, administration of highly species‐specific antibiotics may serve as a potential strategy for cancer treatment.

### 5.2 Dysbiosis of Breast and Gut Microbiomes and Breast Cancer Metastasis

Microbial richness and community diversity in the GI tract reduce from healthy individuals to those with primary breast cancer to those with breast cancer metastases to the bones [17]. Specifically, the levels of Proteobacteria, *Staphylococcus*, *Campylobacter*, and Moraxellaceae are higher and those of *Paraprevotella* are lower in patients without bone metastasis than in healthy individuals. In contrast, levels of Lactobacillales, Bacilli, *Veillonella*, *Streptococcus*, *Campylobacter*, Epsilonproteobacteria, *Acinetobacter*, Pseudomonadales, Moraxellaceae, and *Collinsella* are higher and those of *Megamonas*, Clostridia, *Akkermansia*, *Gemmiger*, and *Paraprevotella* are lower in patients with bone metastases than in healthy individuals. This highlights the dramatic differences in the distribution of microflora in the three physiological conditions. The study also suggests that absence of particular microbes deprives patients of the protective effects of microbiota, which accelerates bone metastasis. Finally, pathway analysis indicated enrichment of sex steroid hormone biosynthesis in patients with bone metastasis [17]. Furthermore, Rosean et al. showed that gut dysbiosis in mice with hormone receptor‐positive breast cancer enhanced levels of circulating cancer cells and metastasis of breast cancer cells to the lymph nodes and lungs. They also observed that dysbiosis led to systemic and local changes in the tumor microenvironment, along with myeloid cell recruitment in the breast tissue and tumor [86]. Taken together, these studies highlight that alterations in the gut microbiome may influence breast cancer metastasis. Some of the gut microbial associations with breast cancer and metastasis have been presented in Table 1.

**TABLE 1:** Association of gut and breast tissue microbiome with breast cancer metastasis.

Microbiome type	Study type	Microorganisms involved	Mechanism	Impact on metastasis	References
Gut microbiome	In vitro and in vivo	*Fusobacterium nucleatum*	Bacterial colonization suppresses accumulation of tumor‐infiltrating T cells and NK cells	Promotes metastasis	[83]
	Clinical	Clostridiaceae, *Faecalibacterium*, Ruminococcaceae	β‐Glucuronidase producing microorganisms convert conjugated inactive form of estrogen to deconjugated biologically active form	Promotes metastasis	[35]
	Clinical	*Bifidobacterium, Blautia, Faecalibacterium, Prausnitzii*	Alteration of the enterohepatic circulation of estrogens and/or the metabolism of phytoestrogens	Not specified	[45]
	In vitro and in vivo	*Staphylococcus xylosus, Lactobacillus animalis*, and *Streptococcus cuniculi*	Enhances resistance to FSS by reorganizing the actin cytoskeleton	Promotes metastasis	[15]
	In vitro and in vivo	*Escherichia coli*	Indole‐propionic acid reduces expression of vimentin, FGFBP1, Snail, and β‐catenin; and upregulates expression of E‐cadherin to suppress epithelial–mesenchymal transition	Inhibition of cancer metastasis	[87]
	In vitro and in vivo	*Escherichia coli*	Cadaverine (produced by the intestinal microbiome) reduces motility and metastatic nature of cancer stem cells by restoring epithelial–mesenchymal transition	Inhibition of cancer metastasis	[88]
	Clinical	*Lactobacillus, Bifidobacterium, Escherichia coli*, and so forth	Regulation of inflammation and immune response	Inhibition of cancer metastasis	[89]
	In vivo	*Streptococcus, Campylobacter* and Moraxellaceae	Steroid hormone biosynthesis by these bacterial species influences bone metastasis	Promotes metastasis	[17]
	In vivo	Antibiotic‐induced commensal dysbiosis	Dysbiosis enhanced levels of circulating tumor cells and metastasis in the lungs	Promotes metastasis	[86]
Breast tissue microbiome	In vitro and in vivo	*Staphylococcus aureus*	*S. aureus* induces autophagy‐dependent neutrophil extracellular traps that increase breast cancer cell metastasis	Promotes metastasis	[90]
	In vitro and in vivo	*Staphylococcus* and *Lactobacillus*	Invasion of tumor cells with bacteria trigger certain changes in tumor cell behavior which include the metastatic property	Promotes metastasis	[15]
	Clinical	*Proteobacteria* spp. and *Listeria* spp.	Influences expression profiles of genes involved in epithelial–mesenchymal transition	Promotes metastasis	[91]
	Clinical	*Bacteroides fragilis*	Breast tumor progression and metastasis through the secretion of the *B. fragilis* toxin (BFT)	Promotes metastasis	[92]
	Clinical, in vitro, and in vivo	*Fusobacterium nucleatum*	*F. nucleatum*‐derived extracellular vesicles enhanced breast cancer cell metastasis via toll‐like receptor 4	Promotes metastasis	[93]

While the direct links between the breast microbiome and bone metastases are poorly understood, it is possible that the breast microbiome may influence the metastatic potential of breast cancer cells and their ability to colonize at distant site, including the bone (Table 1). For instance, intracellular bacteria induce cytoskeletal remodeling that protects circulating breast cancer cells from fluid shear stress [15] and increase cellular motility and ensure cell migration [92]. It is even more interesting to note that such intratumoral bacteria remain alive and harbor in the cytoplasm, rather than the extracellular space. Nejman et al. reported that intratumor bacteria were frequently present inside cancer and immune cells, for example, macrophages were found to harbor 16*S* rRNA and lipopolysaccharide after phagocytosis, suggesting presence of bacterial components intracellularly. The study also suggested that microbiome of the breast tumors was more diverse than that of tumors at other sites [94]. Further, studies have suggested that intracellular bacteria aid in breast cancer metastasis, but not primary cancer growth. For instance, as reviewed by Wang, He, and Wang, treatment of breast cancer with antibiotics was found to reduce the propensity of metastases to the lungs. Specifically, *Staphylococcus*, *Lactobacillus*, and *Streptococcus* were found to help promote breast cancer metastases. Additionally, they reviewed that intracellular bacteria may help in preventing damage to cancer cells during movement through blood vessels, especially by altering the RhoA‐ROCK signaling pathway that aids in cytoskeleton remodeling, this enables cancer cells to sustain their journey to distant organs, including the bone. However, Wang, He, and Wang cautioned that in case of cancers originating in the breast and brain, the origin of intratumoral bacteria warrants more detailed investigation [95]. In contrast to the roles of tissue bacteria, viruses—as part of the tissue microbiome—have varied effects on breast cancer development, progression, and metastasis. For instance, mouse mammary tumor virus (MMTV) is known to cause breast cancer in mice, but Khalid et al. clarified that MMTV‐like virus prevalence does not associate with patient TNM status and breast cancer metastasis [96]. Whereas, Fathy et al. showed that infection with hepatitis C virus correlated with lymph‐node invasion, high tumor grade, and distant metastasis in patients with breast cancer [97]. Additionally, Purrahman et al. have attempted to discuss the role of human papillomavirus with genomic instability, dysfunction of immune system, and distant metastasis of breast cancer [98]. Therefore, it would be preferable to fully understand the mechanisms of how viruses influence breast cancer metastasis, before targeting them for therapeutic purposes.

**FIGURE 1:**
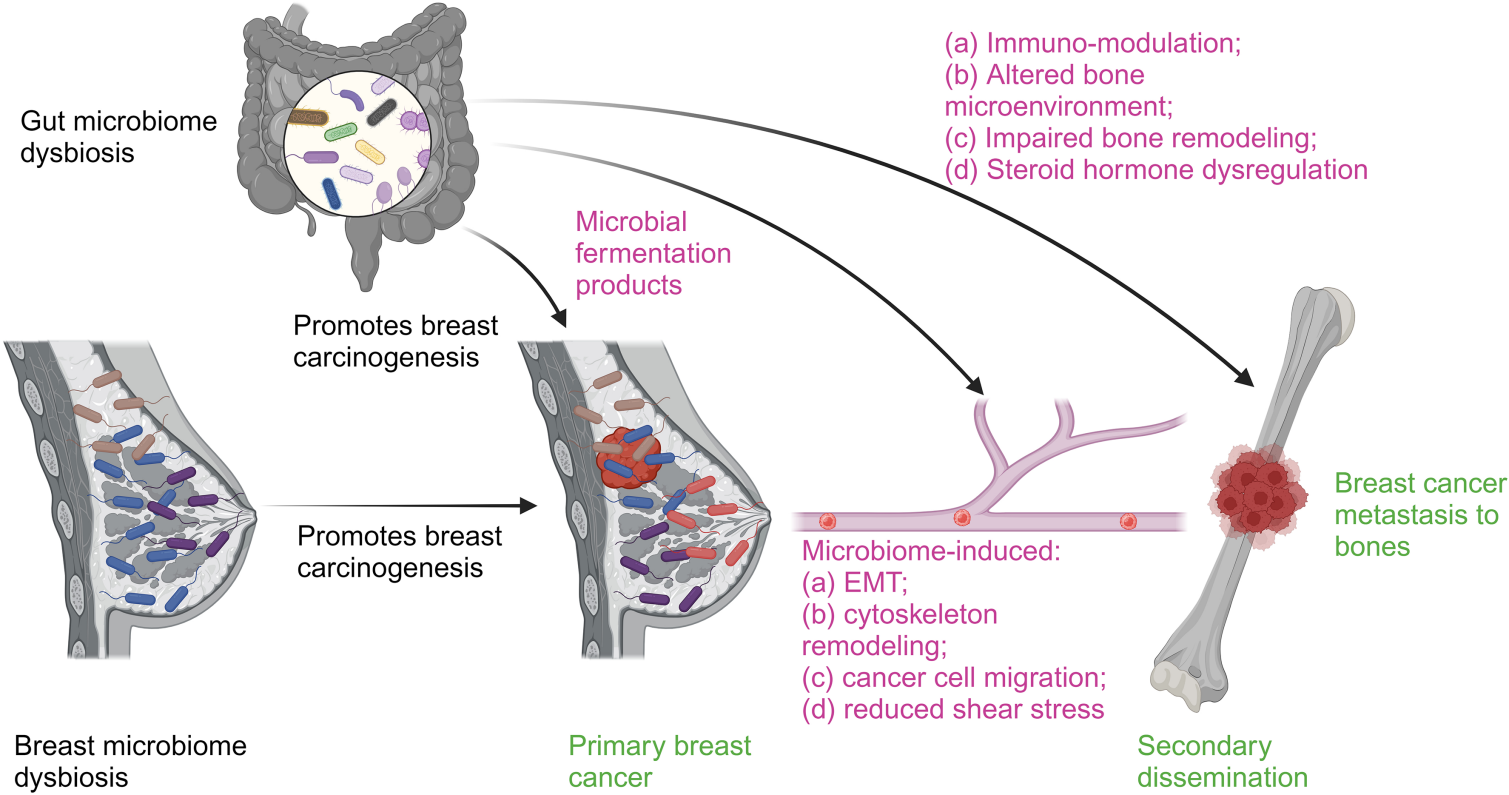
Potential mechanisms employed by gut and breast microbiomes to influence breast cancer metastases to the bone. Figure highlights the multifaceted roles of gut and breast microbiomes in promoting breast cancer metastasis to bones, emphasizing the interplay between microbial communities, cancer, and bone microenvironment. Gut and breast microbiome dysbiosis: An imbalance in the gut and breast microbiomes can lead to the production of certain microbial fermentation products that affect cancer progression. Microbial fermentation products in circulation: These products can promote epithelial–mesenchymal transition (EMT), a critical step in cancer metastasis. EMT: EMT involves changes in cancer cells that enhance their ability to migrate and invade other tissues. Microbial products and microbes themselves play a key role in EMT. Cytoskeletal remodeling: Microbes influence changes in the cytoskeleton of cancer cells that facilitate their migration, invasion, and escape into circulation. Reduced shear stress: Intracellular bacteria reduce effect of shear stress on cancer cells and help their survival in circulation, and thus, cancer cells can eventually invade distant organs, including the bone. Immunomodulation: Microbiome‐induced changes in the immune system can either support or hinder cancer metastasis. Altered bone microenvironment: Microbiome dysbiosis can alter the bone microenvironment, making it more conducive for colonization of cancer cells. Impaired bone remodeling: Disruptions in normal bone remodeling processes, induced by microbial products, can facilitate the establishment of metastases in the bone. Steroid hormone dysregulation: Microbiome dysbiosis can dysregulate steroid hormone levels, further impacting cancer progression and metastasis.

Next, several studies have explained the role of tissue microbiome as driving forces for epithelial–mesenchymal transition (EMT) and cancer cell migration. First, as mentioned above, Fu et al. explained how intratumoral bacteria remain associated with migrating breast cancer cells and aid in formation of metastasis [99]. Second, a study by Qi et al. demonstrated how infection with *Staphylococcus aureus* induces autophagy‐dependent formation of neutrophil extracellular traps that influence metastasis of breast cancer cells to the lungs [90]. Further, microbial composition and tumor‐specific gene expression have been found to correlate in breast cancer. For instance, the presence of *Listeria fleischmannii* correlates with the expression of genes involved in EMT, thus priming human cells for metastasis, whereas, the presence of *Haemophilus influenzae* correlates with the expression of genes associated with regulation of G2‐M checkpoint, E2F signaling, and mitotic spindle assembly [91]. Moreover, Li et al. observed an increased level of genomic DNA of *Fusobacterium nucleatum* in the breast tissue of patients with breast cancer, also, small extracellular vesicles derived from *F. nucleatum* were found to increase breast cancer cell migration, invasion, and proliferation in vitro and metastasis in vivo, via TLR4, suggesting important role of breast tumor microbiota in the progression and metastasis of breast cancer [93]. It is worth noting that *F. nucleatum* has been implicated in tumorigenesis, metastasis, and therapy resistance in multiple cancer types including colorectal, breast and oral cancers, making it a promising therapeutic candidate [100]. A study by Chiba et al. suggested that neoadjuvant chemotherapy led to increased intratumoral levels of *Pseudomonas*, and patients who developed distant metastasis showed enhanced levels of *Staphylococcus* and *Brevundimonas* in the breast tumors. Thus, the study highlighted these intratumoral microbes as potential biomarkers to predict breast cancer metastasis [101]. A more detailed review of different microbes correlating with invasive, migratory, and metastatic phenotype of breast cancer is available elsewhere [102]. Next, presence of microbes has been shown to selectively enrich matrix metalloproteinases that either enhance or reduce cancer metastasis (reviewed in [81]). In contrast, an attenuated strain of *Salmonella typhimurium* has been shown to abrogate the metastasis of breast cancer cells to the bones [103]. While the study provides a proof‐of‐concept, more investigations are required to elucidate the mechanisms by which a pathogen can reduce metastatic activity of breast cancer cells. Figure 1 provides a schematic representation of the myriad mechanisms utilized by gut and breast microbiomes to influence metastasis of breast cancer cells to the bone.

## 6 Strategies for Modulating the Breast and Gut Microbiomes to Prevent or Impede Bone Metastases

Breast cancer cells tend to form micrometastases or inactive dormant disseminated tumor cells in the bone marrow before forming macrometastases. These dormant forms increase the risk of poor overall survival and disease‐free survival (reviewed in [23]). Adjuvant therapies, such as bisphosphonate therapy and chemotherapy, are effective in eliminating bone marrow cells. Bisphosphonate therapy can inhibit osteoclastic bone resorption and prevent bone metastasis in patients with early‐stage breast cancer [104]. In contrast, while standard chemotherapy (with docetaxel, cyclophosphamide, epirubicin, or methotrexate) is effective in clearing rapidly dividing cancer cells, it is ineffective in destroying dormant disseminated tumor cells in the bone marrow [105]. Thus, a treatment that targets interaction between metastatic cells and osteoclasts–osteoblasts–osteocytes (bone remodeling) may aid in prevention or treatment of breast cancer metastases to the bone. Denosumab, an anti‐RANKL human monoclonal antibody, is efficient in preventing skeletal‐related events in patients with breast cancer metastasized to the bone. However, both these agents only marginally improve patient outcomes [106]. In addition, an α‐particle‐emitting radiopharmaceutical drug, radium‐223 dichloride (^223^RaCl_2_), which targets bone metastases of osteoblastic origin, has been shown to only marginally improve survival of patients with bone metastasis [107]. Further, anti‐PD‐1, anti‐PD‐L1, or anti‐CTLA4 monoclonal antibodies have been used as immune checkpoint blockade agents to counter metastatic tumors, although with limited success [108]. However, bone metastases are resistant to immune checkpoint blockade therapy due to enhanced TGF‐β production by their interactions with osteocytes and osteoblasts [109]. Therefore, these agents have limited efficacy in the treatment of bone metastases, necessitating the application of more specific, less toxic therapeutic agents.

**FIGURE 2:**
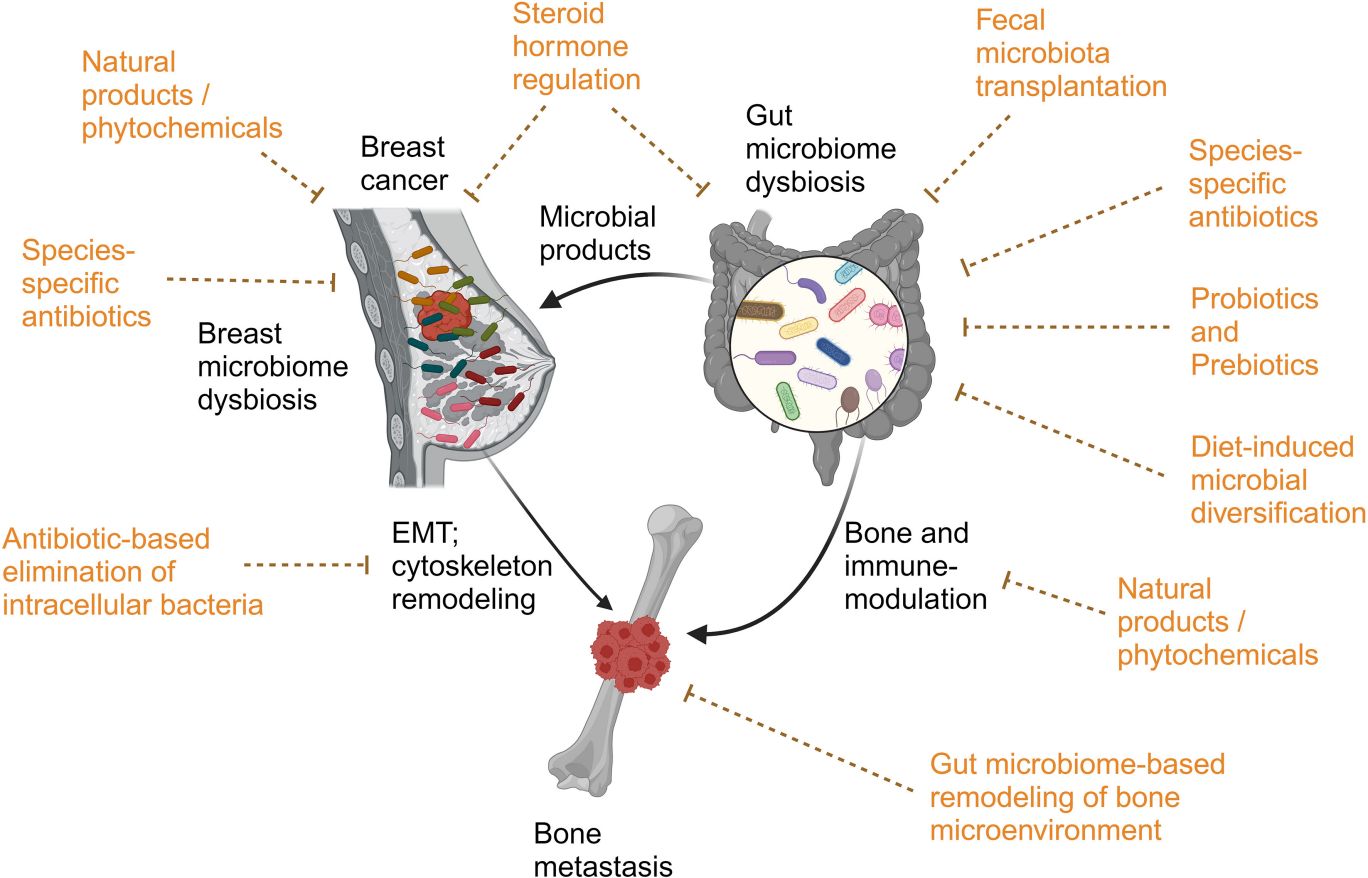
Plausible therapeutic options for targeting gut and breast microbiomes to impede breast cancer metastasis to bones. Figure summarizes some therapeutic options to abrogate breast cancer growth and metastasis, especially to the bone. Fecal microbiota transplantation (FMT): Transferring gut microbiota, or a concoction of specific microbial species, from healthy donors and/or therapy‐responders into patients with cancer and/or therapy‐nonresponders, may help in restoring a balanced microbiome, which may potentially impede cancer progression and metastasis. Species‐specific antibiotics: Administration of antibiotics that specifically target pathogenic microbial species in the gut and breast microbiomes could reduce their impact on cancer progression. Dietary inclusion of probiotics and prebiotics: Probiotics can help ensure a healthy microbiome, reducing dysbiosis and potentially abrogating cancer growth and metastasis. Prebiotics promote the growth of beneficial microbes, which may help in preventing cancer metastasis. Dietary inclusion of phytochemicals: Certain plant‐derived compounds can modulate the microbiomes and directly or indirectly inhibit cancer progression and metastasis. Steroid hormone regulation: Regulating metabolism of steroid hormones through microbiome‐targeted therapies could mitigate their role in cancer progression and metastasis to the bone. Gut microbiome‐based remodeling of bone microenvironment: Modulating the gut microbiome to influence the composition of the bone microenvironment may prevent the establishment and growth of metastatic cancer cells in bone tissue. Although plausible, some of these therapeutic options are tested for other cancer types, and thus, warrant detailed investigations in case of breast cancer metastasis to the bone.

We have previously described that gut microbiome can either enhance or reduce the anticancer efficacy of agents targeting breast cancer [39]. Therapeutic options for preventing or suppressing bone metastasis in patients with breast cancer are poorly studied. However, some plausible mechanisms deserve to be mentioned. For instance, free fatty acid receptors—FFAR2 and FFAR3—have been shown to respond to SCFAs leading to enhanced E‐cadherin levels and reduction in ERK phosphorylation, which together inhibit EMT of breast cancer cells. Moreover, lithocholic acid, a secondary bile acid, has been shown to activate TGR5, and thus, suppress EMT and angiogenesis in breast cancer (reviewed in [39]). Here, we propose that strategies, including administration of antibiotics or probiotics, may be devised to suppress deconjugation and metabolism of steroid hormones (e.g., estrogen) by gut microbes, and thus, inhibit growth of hormone receptor‐positive breast cancer and its metastasis. Similarly, certain microbial metabolites can be exploited for their antimetastatic activities. For example, urolithin A, secreted by *Enterococcus faecium* FUA027, induces actin depolymerization that reduces cancer cell migration in other cancer types (as reviewed by [110]). Interestingly, 3′‐azido‐3′‐deoxythymidine‐based antibacterial agents have been tested, in vitro, for their anti‐migratory effects on breast cancer cells [111]. Furthermore, narasin, an ionophore‐specific antibiotic, has been shown to inhibit migration and metastasis of estrogen receptor‐positive breast cancer cells in in vitro and in vivo model systems [112]. Tyagi and Patro have elucidated that salinomycin can also prevent proliferation and metastasis of estrogen receptor‐positive breast cancer cells by suppressing the NF‐κB pathway [113]. These agents seem promising as they have dual properties and can also be synthesized for particular microbes (Figure 2).

In other cancer types, modification of the gut microbiome has been found to help improve the antimetastatic efficacy of immune checkpoint inhibitors. Studies recommend administration of probiotics that improve the levels of immune effector T cells and lower levels of immunosuppressor T cells, along with immune checkpoint inhibitors, to reduce bone metastasis [108]. In general, probiotic administration has been found to influence the bone characteristics. For instance, administration of a single probiotic or a mixture of *Lactobacillus* and *Bifidobacterium* has been shown to reduce inflammation of the bone and suppress bone loss in animal models (as reviewed by Parvaneh et al. [114]). Peng et al. have reviewed that probiotics can regulate RANKL and CD4^+^ T cell activity, proinflammatory cytokine levels and osteoblast activity, in addition to regulating levels of SCFAs that strengthen calcium absorption [58]. Moreover, probiotics have been shown to attenuate carcinogenesis, decrease tumor volume, and inhibit metastasis and angiogenesis (reviewed in [115]). For example, *Lactobacillus casei* probiotic (kefir) has been shown to improve levels of T helper and T cytotoxic cells and reduce breast cancer metastases to the lungs and bones [116]. Therefore, it is imperative to select the correct probiotics with the most appropriate concoction of bacteria that may help improve the levels of antitumor immune cells in the host, and hence, enhance efficacy of immune checkpoint inhibitors against bone metastasis. Similarly, prebiotics (e.g., inulin) may be considered as a treatment option for cancer metastasis [117], but detailed studies to test the effects of prebiotics on breast cancer metastasis to the bone remain to be performed. Moreover, some microbial metabolites, such as indole‐propionic acid and lithocholic acid, have been shown to reduce proliferation, aggressiveness, and metastasis of breast cancer [87, 118]. It would be interesting to investigate whether such microbial components can be used for preventing metastasis of breast cancer to the bone (Figure 2).

Further, FMT is a promising therapeutic option for cancers. FMT has multiple potential applications, including improving efficacy of chemotherapy and immunotherapy, prevention and management of cancers, improving host immunity, and so forth. (as reviewed by [119]). Interestingly, a randomized phase‐II clinical trial by de Clercq et al. suggested that allogenic FMT in patients with metastatic gastroesophageal cancer improved response to first‐line chemotherapy and patient survival [120], this highlights the applicability of FMT for treatment of metastatic cancers (Figure 2). In case of breast cancer, Di Modica et al., for the first time, showed that FMT from mice that responded to trastuzumab to mice bearing HER2+ breast cancer improved the effectiveness of trastuzumab [121]. While FMT has been successfully applied for the treatment of multiple cancer types, such as improving efficacy of PD‐1‐based immunotherapy for epithelial tumors and melanoma [43, 122], its widescale utilization for the treatment of metastatic breast cancer remains unexplored [123].

Finally, nutrition plays an important role in the overall development of organisms, as malnutrition leads to severe weight loss and disruption of the gut microbiota [124]. Thus, ensuring adequately balanced, nutritional diet may help prevent gut microbiome dysbiosis or health imbalance, ensure bone health, and possibly prevent alterations of bone architecture (Figure 2). Specifically, diet modification from high fat, high sugar to Mediterranean and Japanese diet has been recommended for improving the gut microbiome composition [125]. Interestingly, the original Indian subcontinental diet is a classic example of a diverse and healthy diet that helps ensure diversity in the gut microbiota, and hence, reverse or prevent multiple chronic diseases [126, 127]. There is currently no conclusive evidence to suggest that Indian diet can reverse cancers, hence, the effects of Indian diet on cancer need to be elucidated in details. Furthermore, the use of natural food/beverage ingredients, such as nanovesicles from edible tea flowers, has been claimed to modulate gut microbiota composition and inhibit in vivo metastasis of breast cancer cells to the lungs [128]. Moreover, Li et al. have extensively reviewed the anticancer and antimetastatic efficacy of several natural products on breast cancer, such as soy, vegetables, fruits, spices, edible micro‐fungi, and cereals [129]. However, a thorough understanding is warranted before implementation of such diet‐based therapies for the treatment of breast cancer and its metastasis to the bone.

## 7 Conclusions

Breast cancer metastasis, especially to the bone, is a serious health concern. This review elaborated on the association between microbiome and breast cancer, and highlighted that the gut and breast microbiomes influence the growth and metastasis of breast cancer to distant organs including the bone. We believe that the review would facilitate discussion and detailed investigations on this health concern. The review provides a detailed overview of the currently understood mechanisms by which breast tissue and gut microbiomes promote metastasis; we believe that these mechanisms may function together or independent of the mechanisms known previously involving epithelial cancer cells. Finally, we have discussed the currently available treatment modalities to treat bone metastases by targeting the gut and breast tissue microbiomes. We believe that targeting specific components may aid in preventing and reducing the propensity of breast cancer metastasis to bones. Specifically, ensuring a diverse gut and breast microbiota with minimal use of broad‐spectrum antibiotics, inclusion of a healthy diet, combinatorial treatment with probiotics and/or prebiotics with anticancer therapeutics, and fecal microbiota transplantation may help in maintaining a diverse gut microbiome, which may aid in preventing breast cancer metastasis.

## Author Contributions

**Amruta Naik:** conceptualization, writing – original draft, writing – review and editing. **Mukul S. Godbole:** visualization, writing – review and editing, writing – original draft.

## Conflicts of Interest

The authors declare no conflicts of interest.

## Data Availability

Data sharing is not applicable to this article as no new data were created or analyzed in this study.
